# Importance of adapted digestion conditions to simulate in vitro lipid digestion of broilers in different life stages

**DOI:** 10.1016/j.aninu.2022.09.008

**Published:** 2022-10-04

**Authors:** Daphne Michels, Sarah H.E. Verkempinck, Agnese Panozzo, Karen Vermeulen, Marc E. Hendrickx, Liesbet Thijs, Tara Grauwet

**Affiliations:** a Laboratory of Food Technology and Leuven Food Science and Nutrition Research Centre (LFoRCe), Department of Microbial and Molecular Systems (M^2^S), KU Leuven, Leuven, 3001, Belgium; b Kemin Animal Nutrition and Health, Kemin Europa N.V., Herentals, 2200, Belgium

**Keywords:** Lipolysis, Lipase activity, Bile salt, In vitro digestion, Broiler chicken, Emulsion

## Abstract

In vitro digestion studies demonstrate large potential to gain more and quicker insights into the underlying mechanisms of feed additives, allowing the optimization of feed design. Unfortunately, current in vitro digestion models relevant for broiler chickens lack sufficient description in terms of protocols and standardisation used. Furthermore, no distinction is made between the different life phases of these animals (starter, grower, and finisher). Hence, our research aimed to establish adapted in vitro digestion conditions, corresponding to the 3 life phases in broilers, with specific focus on lipid digestion. The effect of 3 different bile salt concentrations of 2, 10, and 20 mM, and 3 different lipase activities of 5, 20, and 100 U/mL, on in vitro lipid digestion kinetics were evaluated using a full factorial design. These values were selected to represent starter, grower, and finisher birds, respectively. Our findings showed that the extent of lipid digestion was mainly influenced by lipase activity. The rate of lipid digestion was affected by an interplay between bile salt concentration and lipase activity, due to possible lipase inhibition at certain bile salt concentrations. Overall, this work resulted in 3 in vitro lipid digestion models representative for starter, grower, and finisher birds. In conclusion, this research showed the impact of adapted in vitro digestion conditions on lipid digestion kinetics and thus the need for these conditions relevant for each life phase of broilers.

## 1 Introduction

Poultry meat is currently the most consumed type of meat with a global annual production of 135.4 million tonnes in 2021 (FAO, 2021). In the past years, increasing production and consumption was reported by the Food and Agriculture Organisation (FAO, 2020, 2019), leading to a continuous need for optimization of feed and feed additives to meet current and future needs sustainably.

The effectiveness of feed additives in animal production is mostly evaluated by means of in vivo experiments. In vivo studies are still considered “the gold standard” as they immediately provide information regarding the potential of the feed additive, and results can be immediately interpreted and applied. However, disadvantages exist such as ethical constraints, high cost, high demand of resources, and lack of standardised results due to large variability. Furthermore, in vivo data often fail to elucidate the mechanism driving the effect of a certain additive as mainly endpoint measurements are evaluated (Bornhorst et al., 2016; Mota de Carvalho et al., 2021)*.* These latter aspects can be complimented by the potential of in vitro testing.

The major advantages of performing in vitro digestion studies are its high throughput, low cost, lack of ethical hurdles, and its potential for standardisation, as proven by multiple studies within food technology (Bornhorst et al., 2016; Zaefarian et al., 2021). Moreover, taking samples as a function of the digestion process from broilers in vivo is a less approachable practice, while in vitro digestion studies allow the assessment of the evolution of the digestion process (e.g. macronutrient hydrolysis patterns, food structural changes) throughout the entire digestive process (Bornhorst et al., 2016). Furthermore, multiple studies within food technology have demonstrated the correlation between in vitro and in vivo data, illustrating that in vitro studies are a highly useful tool to study digestion extents and how food design can impact (macro)nutrient digestibility (Bohn et al., 2018; Dupont et al., 2019).

While in vitro digestion approaches have been already used in the broiler feed technology field, in vitro conditions were often poorly adapted to the conditions of the broiler gastrointestinal tract (GIT) and differed from study to study. Thus, one problem faced is the lack of standardisation within the current in vitro digestion models for broilers, resulting in contradictory results (Zaefarian et al., 2021). A standardised protocol would facilitate the comparison of results from different research institutes (Mota de Carvalho et al., 2021). Another problem is that these in vitro digestion models are often based on in vitro models simulating the human digestive tract. This is a good base due to large resemblances yet, authors frequently do not explain which adaptations were made to the model, nor do they sufficiently adapt the model to fit the broiler GIT (Boisen and Eggum, 1991; Bosc-Bierne et al., 1984; Herwig et al., 2019; Jimenez-Moya et al., 2021; Mota de Carvalho et al., 2021; Ravindran, 2013; Svihus, 2011). Previous research showed the importance of selecting the right set of physiologically relevant parameters to simulate the intended gastrointestinal tract as they will influence the digestive process (Bornhorst et al., 2016; Dupont et al., 2019). Therefore, different sets of physiological parameters have to be selected in order to more appropriately simulate the digestive tract of different populations in vitro.

Diets for broiler chickens are formulated to meet their nutritive requirements for each phase of the grow-out period, starter, grower, and finisher phase, as their GIT develops during their lifetime (Pope et al., 2004; Warren and Emmert, 2000). This paper will specifically focus on lipid digestion in broilers, as lipids are an important, high caloric nutrient routinely added to broiler diets to meet specified energy densities. Nevertheless, research showed that especially young broilers have difficulties to digest lipids as secretions of bile salts and lipase are rather low in starter birds, but increase as birds grow (Krogdahl, 1985). To solve this problem, emulsifiers are frequently added as a feed additive to improve lipid digestion (Coon, 2002; Siyal et al., 2017). To evaluate the effectiveness of these feed additives in vitro, this paper aimed to evaluate conditions relevant for the 3 life stages of broilers with specific focus on lipid digestion.

To the authors’ knowledge, no standardised parameters to simulate in vitro lipid digestion of broilers, taking into account the heterogeneity of conditions taking place during their different life phases, have been developed. As per previous in vitro digestion studies aiming to simulate the broiler GIT, a human protocol was used as a starting point. We selected the widely used INFOGEST protocol (Brodkorb et al., 2019). This human model was selected as a relevant starting point for in vitro simulation as factors such as pancreatic lipase, digestion time, and pH values are similar to those of chickens (Bosc-Bierne et al., 1984; Ravindran, 2013; Svihus, 2011). To evaluate the need for different digestive conditions for each broiler life phase, the effect of increasing bile salt concentrations and lipase activities on lipid digestion kinetics was evaluated.

## 2 Materials and methods

### 2.1 Animal ethics and materials

This study only contains in vitro work, and no animals were used. Soy oil and a soy lysolecithin-based surfactant were supplied by Kemin Europa (Herentals, Belgium). Soy oil was transferred to amber glass bottles, the headspace was flushed with nitrogen, and bottles were stored at −20 °C until use. Pancreatic extract was donated by Nordmark (Uetersen, Germany) and had a lipase activity of 125 U/mg (data not shown). Analytical and HPLC grade reagents were purchased from Sigma–Aldrich (Diegem, Belgium), besides NaHCO_3_, NaCl, H_2_SO_4_, KH_2_PO_4_, ethanol and trimethylamine (Fisher Scientific, Merelbeke, Belgium); KCl, MgCl_2_(H_2_O)_6_ and heptane (Acros Organics, Geel, Belgium); HCl, diethylether and iso-propanol (VWR, Leuven, Belgium); and lipid standards (Larodan, Solna, Sweden).

### 2.2 Oil-in-water emulsion preparation

Oil-in-water (20%) emulsions were prepared by first dissolving the lysolecithin-based surfactant in soy oil (surfactant-to-oil ratio = 0.2) in a water bath at 40 °C. Next, a coarse emulsion was prepared by mixing the oil/surfactant mixture with demineralized water (ratio 12:50, vol:vol) at 10,000 rpm for 5 min (Ultra-Turrax T25, IKA, Staufen, Germany). The coarse emulsion was further homogenized at 20 MPa (1 cycle) with a high-pressure homogenizer (STANSTED SPCH-10, Homogenising Systems, Harlow, UK). Emulsions were prepared and used within one day.

### 2.3 In vitro digestion conditions relevant for different life phase of broilers

As explained in the introduction, we aimed to evaluate the effect of a range of in vitro digestion conditions relevant for different broiler life phases on lipid digestion kinetics. For this purpose, the standardized INFOGEST protocol relevant for human digestion was used as a starting point (Brodkorb et al., 2019). More specifically, a scaled-down version of this protocol was followed (Infantes-Garcia et al., 2021). Some adaptations were applied to this protocol to obtain protocols mimicking the GIT of broilers at 3 stages of their lifetime. As broilers immediately swallow the pecked feed and the use of the crop is rather negligible in broilers fed ad libitum, these phases were not simulated in the current study (Parkhurst and Mountney, 2012; Svihus, 2014). However, it is important to note that the crop is still used in chickens fed ad libitum, but it is strongly believed that most feed surpasses the crop, immediately entering the proventriculus and gizzard (Classen et al., 2016). Moreover, no lipid digestion takes place in the crop Therefore, the relevance of crop simulation for our work was estimated very low (Ravindran et al., 2016).

Briefly, the gastric phase was simulated by mixing 250 μL oil-in-water emulsion, 200 μL simulated gastric fluid, 5 μL CaCl_2_ (7.5 mM), 41.5 μL milli Q water, and 3.5 μL HCl (0.6 M) (to adjust the pH to 3). The proventriculus of broilers does not secrete gastric lipase (Krogdahl, 1985). Even though pancreatic lipase can enter the gizzard due to duodenal refluxes, it is hypothesized that only a small fraction of lipids can be digested there due to unfavourable conditions including low pH. At pH values lower than 5, which are typical for the proventriculus/gizzard, pancreatic lipase is inactivated. Hence, the major part of lipolysis takes place in the small intestine (Duke, 1992; Rodriguez-Sanchez et al., 2019). Thus, nor gastric or pancreatic lipase was considered when simulating the gastric phase. The headspace of the tubes was flushed with nitrogen gas after which samples were rotated end-over-end in an incubator at 37 °C for 2 h. Next, the small intestinal phase was simulated by adding 200 μL of simulated intestinal fluid, 40 μL CaCl_2_ (7.5 mM), 125 μL pancreatin solution, 75 μL bile salt solution, 58 μL milli Q water, and 2 μL NaOH (500 mM) (to adjust pH to 7). Once more, the headspace was flushed with nitrogen and samples were rotated end-over-end in an incubator at 37 °C for 2 h. Bile salt concentrations and pancreatic lipase activities were adapted to 2, 10, and 20 mM and 5, 20, and 100 U/mL chyle respectively to simulate small intestinal conditions of the starter, grower, and finisher phase of broilers (Green and Kellogg, 1987; Krogdahl, 1985; Noy and Sklan, 1995; O'Sullivan et al., 1992). To gain a full understanding of the impact of each factor (i.e. enzyme activity and/or bile salt concentration) on the lipid digestion rate and extent, a full factorial designed experiment was conducted, resulting in 9 different testing combinations (Table 1).

**Table 1:** The 9 test combinations (TC) of bile salt concentrations and lipase activities studied in the context of in vitro lipid digestion simulation in broilers considering their different life phases.

TC^1^	Bile salt concentration, mM	Lipase activity, U/mL chyle
1	2	5
2	2	20
3	2	100
4	10	5
5	10	20
6	10	100
7	20	5
8	20	20
9	20	100

1 Test combination 1, 5, and 9 represent the actual conditions which are most relevant for simulating the gastrointestinal tract of starters, growers, and finishers respectively.

Independent samples were taken at consecutive timepoints (0, 5, 10, 15, 20, 30, 45, 60, 90, and 120 min) to analyse lipid digestion kinetics in the small intestinal phase. Digestion was simulated using individual tubes for separate timepoints instead of sampling out of one digestion container. The consecutive, independent timepoints belong to the same digestion, thus they have to be statistically analysed together to evaluate the digestion kinetics. This means that in this context, the individual timepoints can statistically be seen as repetitions (Section 2.6), thereby guaranteeing repeatability of the digestions (Pälchen et al., 2021; Verkempinck et al., 2018).

### 2.4 Quantitative analysis of lipid digestion products

Firstly, lipid species were extracted from all digests as described by Infantes-Garcia et al. (2021). Briefly, 3 mL of diethylether-heptane (1:1, vol:vol) solution, 2 mL of ethanol, and 0.200 mL of sulfuric acid (2.5 M) was added to the digest (1 mL). This mixture was vortexed for 2 min and subsequently mixed for 30 min using an end-over-end rotator (15 rpm). Hereafter, the upper (non-polar) phase was transferred to a 5-mL flask and 1 mL of diethylether-heptane (1:1, vol:vol) solution was added to the lower phase, which was then vortexed again for 2 min and rotated end-over-end for 15 min. Next, the upper phase was collected in the 5 mL volumetric flask again, which was then further diluted to 5 mL with diethylether-heptane (1:1, vol:vol) solution. These extracts were then filtered (Chromafil PET filters, 0.20 μL pore size, 25 mm diameter) and stored in amber glass vials at −80 °C until analysis (maximally 1 week).

Secondly, lipid extracts were analysed using the lipid quantification method described by Guevara-Zambrano et al. (2022). Briefly, samples were injected in an HPLC system (1200 Series, Agilent Technologies, Diegem, Belgium) equipped with a silica column (Chromolith Performance Si, 100 mm × 4.6 mm, Merck, Darmstadt, Germany) and a charged aerosol detector (Corona Veo, Thermo Fisher Scientific, Geel, Belgium). The charged aerosol detector was operated at an evaporation temperature of 35 °C and a nitrogen gas pressure of 5.5 bar (1 bar = 0.1 MPa). An external oven (Chromaster 5310, VWR, Hitachi Ltd., Tokyo, Japan) was used to maintain a column temperature of 40 °C. Lipid digestion products (i.e. free fatty acids [FFA], monoacylglycerols [MAG], diacylglycerols [DAG], and triacylglycerols [TAG]) were eluted by means of a quaternary gradient program using dichloromethane (solvent A), acetonitrile (solvent B), isopropanol-water solution (85:15, vol:vol) containing 0.05% (vol:vol) of acetic acid and triethylamine (solvent C), and isopropanol (solvent D). Next, FFA, MAG, DAG, and TAG were quantified using oleic acid, monoolein, diolein, and triolein as lipid standards, respectively. Subsequently, lipid hydrolysis (%) was calculated based on all lipid reaction products. Equation (1) represents the percentage of lipid hydrolysis as the ratio of hydrolysed bonds over the total amount of hydrolysable bonds.

(1) $$\mathrm{Lipid}\;\mathrm{hydrolysis}\;\left(\%\right)=\left[\mathrm{concentration}\;\mathrm{FFA}\;\mathrm{in}\;\mathrm{digest}\;\left(\frac{\mathrm{\mu mol}}{\mathrm{mL}}\right)\right]/\left[\mathrm{concentration}\;\left(3\times \mathrm{TAG}+2\times \mathrm{DAG}+1\times \mathrm{MAG}+\mathrm{FFA}\right)\;\mathrm{in}\;\mathrm{digest}\;\left(\frac{\mathrm{\mu mol}}{\mathrm{mL}}\right)\right]\times 100$$

### 2.5 Microstructural characterization

The microstructure of the emulsion and selected digests (after 60 and 120 min in the gastric phase and after 0, 60, and 120 min in the small intestinal phase) was evaluated to verify whether changes in particle size occurred as a result of the different digestion conditions applied. Such changes might affect lipid digestion kinetics (McClements, 2018). Microstructural analysis was performed for the 2 most distinct test combinations (TC, i.e. 1 and 9).

Firstly, the particle size (distribution) was measured using laser diffraction (Laser Diffraction Particle Size Analyzer LS 13320 (Beckman Coulter Inc., Indianapolis, IN, USA). Briefly, a few drops of sample are added to a demineralized water tank in which the sample is pumped (speed 30%) to the measurement cell. Herein, laser light (wavelength 750 nm; wavelengths halogen light for polarization intensity differential scattering [PIDS] of 450, 600, and 900 nm) is scattered because of passing particles. The resulting intensity of the diffracted light is measured and transformed according to the Mie theory resulting in volume- and surface area-based mean diameters, d (4, 3) and d (3, 2) in micrometres, respectively. The volume-based mean is more prone to the presence of larger particles, making it a better indicator to evaluate emulsion stability. Thus, it was opted to report also the surface area-based mean since this value is more relevant in the context of studying digestion kinetics as components such as bile salts and lipase adsorb to the oil-in-water interface.

Secondly, the microstructure of the emulsion and digests were visualized using light microscopy (Olympus BX-51, Olympus Optical Co. Ltd., Tokyo, Japan, equipped with a XC-50 digital camera).

### 2.6 Statistical analysis

Lipid digestion kinetic data were statistically analysed and modelled using the statistical software program JMP (JMP Pro15, SAS InstituteInc., Cary, NC, USA). The kinetic behaviour was analysed by single-response, non-linear regression modelling using an empirical fractional conversion model described in Eq. (2):

(2) $$C=C_{f}\times \left(1-e^{-k\times t}\right)\;,$$

where *C* is the extent of lipid hydrolysis (%) at digestion time *t*, *C*_*f*_ is the plateau value of lipid hydrolysis at an infinite time (%), and *k* is the rate constant of lipid hydrolysis (min^−1^). Significant differences among the kinetic parameters estimated were compared using their corresponding 95% confidence intervals.

Furthermore, the potential relation between the estimated kinetic parameters (*C*_*f*_, *k*) and digestion variables (bile salt concentration, lipase activity) was evaluated through correlation plots. The degree of correlation between all parameters was then evaluated by fitting a trendline and assessing *R*^2^.

Lastly, the significant impact of bile salt concentration and lipase activity on the estimated rate constant *k* and the final extent *C*_*f*_, respectively, were evaluated using multiple linear regression with backward elimination (SAS version 9.4, SAS Institute, Inc., Cary, NC, USA). This resulted in models explaining the relation between the responses (*k* and *C*_*f*_) and the explanatory variables (bile salt concentration and lipase activity, their interaction term, and their squares). The fit of the model was assessed by *R*^2^.

## 3 Results

### 3.1 Microstructure of digests in the gastric and small intestinal phase of broilers

**Fig. 1:**
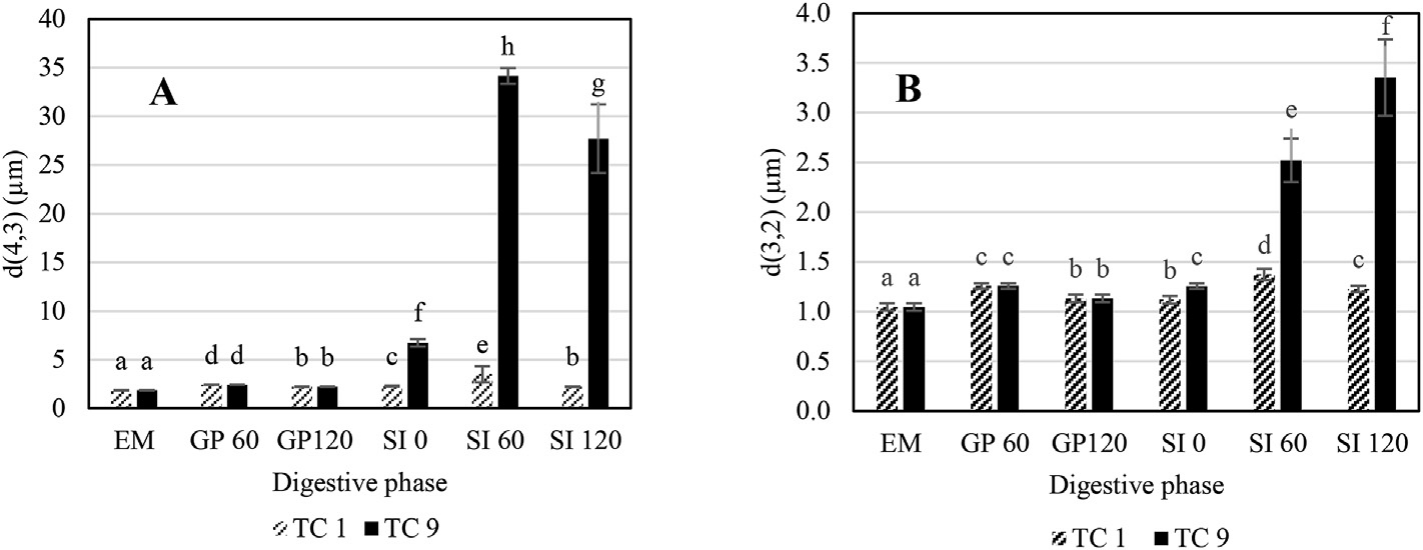
Change in (A) volume and (B) surface area mean particle size of the emulsion (EM), digests in the gastric phase (GP; at 60 and 120 min) and digests in the small intestinal phase (SI; at 0, 60, and 120 min) for test combinations 1 and 9 (TC 1 and 9; Table 1). Bars with different letters report significant differences in mean particle size, *P* < 0.05.

**Fig. 2:**
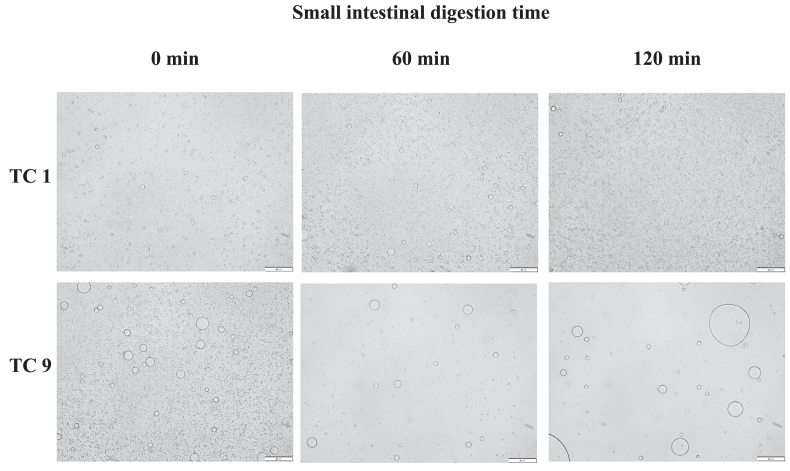
Microstructure of emulsions during digestion. Microscopic pictures of digests in the small intestinal phase (0, 60, and 120 min) of test combination (TC) 1 and 9. Scale bar represents a length of 50 μm.

Microstructural characterisation of TC 1 and 9, which represent digestion conditions for starter and finisher birds, are shown in Fig. 1. These evaluations were performed by means of mean particle size and microscopic evaluations of the emulsions and digests of predetermined timepoints in the gastric and small intestinal phase. First of all, it can be observed that mean droplet size slightly increased as a result of gastric conditions, but overall, the emulsions remained stable. Since the simulated gastric phase was identical for all simulated digestions, the initial microstructure at the beginning of small intestinal digestion was considered identical for all TC (micrographs not shown). In contrast to the gastric phase, mean particle sizes did differ between TC 1 and TC 9 in the small intestinal phase. Besides, microscopic images as part of the small intestinal phase, shown in Fig. 2, confirmed that oil droplets of TC 9 were indeed larger in comparison to TC 1 throughout the small intestinal phase.

### 3.2 In vitro lipid digestion kinetics in the small intestinal phase of broilers

**Fig. 3:**
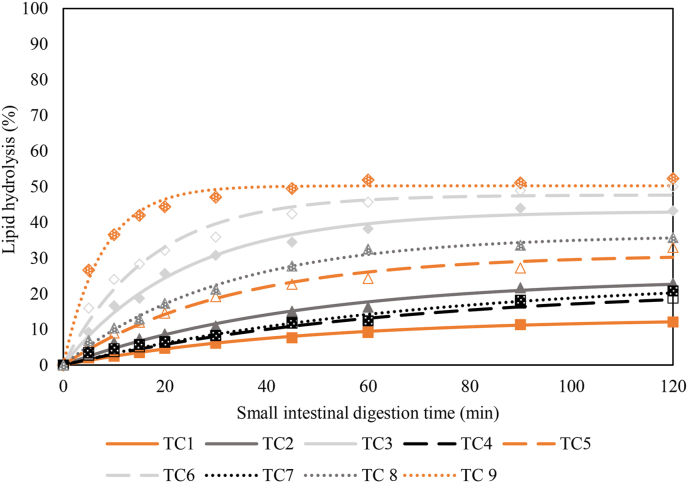
Lipid digestion kinetics expressed in percentage of lipid hydrolysis for all 9 test combinations (TC) (Table 1). Symbols represent experimental data: (▪) 5, () 20, and () 100 U lipase activity per millilitre chyle. Fillings represent (full) 2, (empty) 10, and (speckled) 20 mM bile salt concentrations. Lines represent modelled data according to a fractional conversion model. Digestion conditions representing the starter (TC 1), grower (TC 5), and finisher (TC 9) phase are indicated in orange.

The obtained lipid hydrolysis profiles of the 9 TC (Table 1) are depicted in Fig. 3. Estimated kinetic parameters rate constant *k* (min^−1^) and final extent *C*_*f*_ (%), are shown in Table 2. Furthermore, the analytical method used also allowed to determine all lipid species during digestion and thereby gain more insights into the lipid digestion process (Fig. A, Supplementary Data).

**Table 2:** Estimated kinetic parameters according to a fractional conversion model of the 9 test conditions.

TC^1^	*k* ± SD^2^, min^−1^	*C*_*f*_ ± SD^3^, %
1	0.021 ± 0.001^b^	13.17 ± 0.42^a^
2	0.021 ± 0.002^b^	24.58 ± 1.27^b^
3	0.042 ± 0.003^d^	43.21 ± 1.08^e^
4	0.015 ± 0.005^a^	21.78 ± 3.64^b^
5	0.031 ± 0.003^c^	31.05 ± 1.37^c^
6	0.057 ± 0.008^e^	47.74 ± 1.45^f^
7	0.014 ± 0.003^a^	24.62 ± 2.72^b^
8	0.032 ± 0.002^c^	36.49 ± 0.86^d^
9	0.130 ± 0.009^f^	50.32 ± 0.81^g^

TC = test combination.

^a–g^ Different superscripts indicate significant differences among the different test combinations, *P* < 0.05.

1 Different TC represent different combinations of bile salt concentrations (2, 10, and 20 mM) and lipase activities (5, 20, and 100 U/mL).

2 The *k* is the reaction rate constant of lipid hydrolysis expressed in min^−1^.

3 C_*f*_ is the final estimated amount of lipid hydrolysis expressed as a percentage.

These data demonstrate significant differences in lipid hydrolysis behaviour among different combinations of bile salt concentration and lipase activity. On the one hand, *C*_*f*_ significantly increased as a result of both increasing bile salt concentration and lipase activity, with *C*_*f*_ increasing from 16.70% to 56.58% within the digestive conditions simulated. However, lipid hydrolysis was incomplete in all conditions. On the other hand, rate constant *k* was not always influenced by altering the digestion parameters. Overall, it significantly increased with increasing lipase activities. However, the constant *k* decreased with increasing bile salt concentrations at low lipase activity (5 U/mL), while it increased at high lipase activity (100 U/mL). At intermediate lipase activity (20 U/mL), *k* was not significantly different at various bile salt concentrations. Thus, the data clearly demonstrated significant differences in digestion kinetics (*k, C*_*f*_) between lipid digestion conditions relevant for starters, growers, and finishers.

**Fig. 4:**
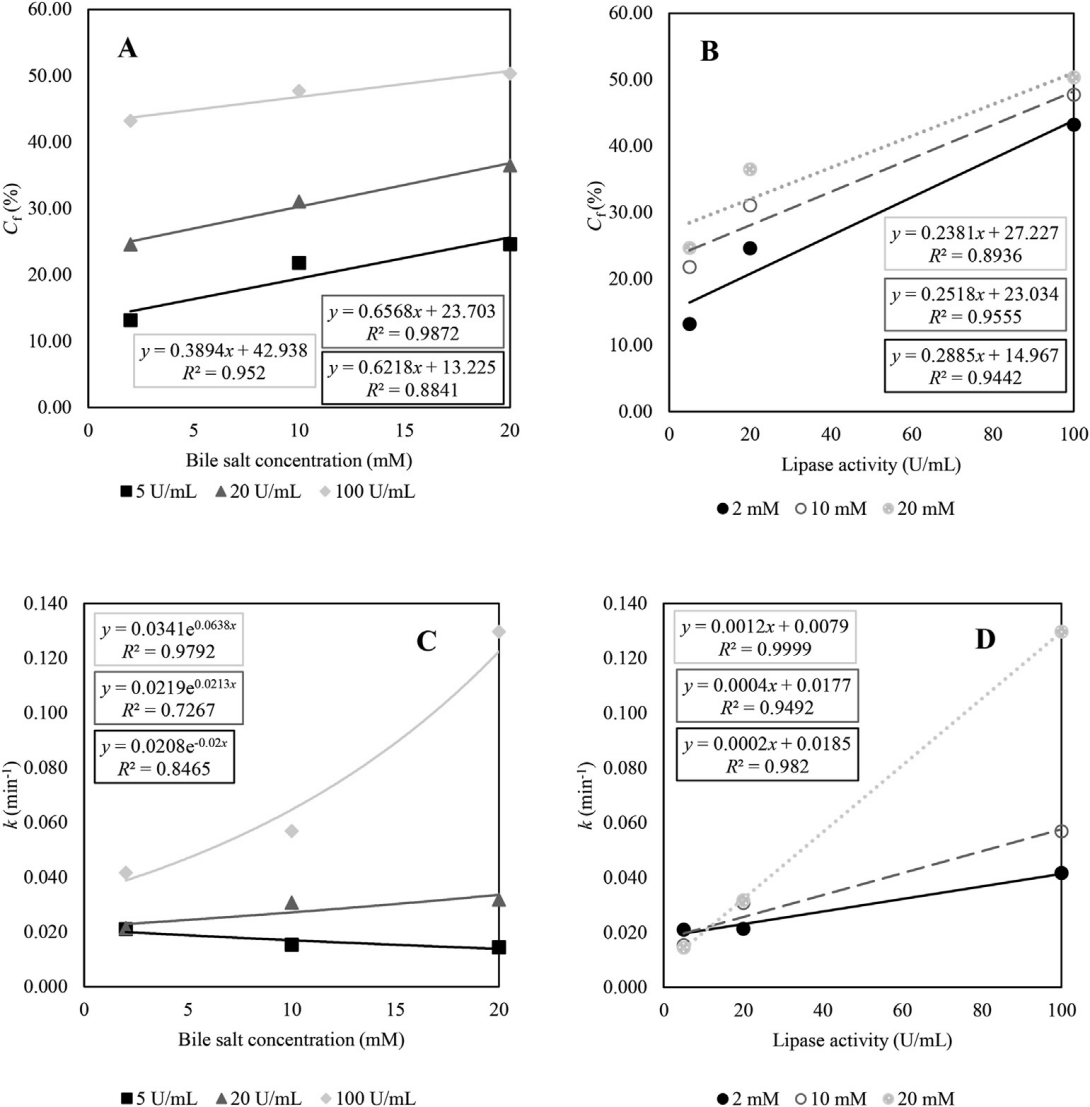
Relation between estimated digestive parameters and digestive conditions. Estimated parameters *C*_*f*_ (final extent, %; graph A and B) and *k* (reaction rate constant, min^−1^; graph C and D) plotted against varied digestive conditions of bile salt concentration (mM; graph A and C) and lipase activity (U/mL; graph B and D). Trendlines equations and corresponding *R*^2^ are reported for each condition. Symbols represent experimental data: (▪) 5, () 20, and () 100 U lipase activity per millilitre chyle, respectively, and (•) 2, (○) 10, and () 20 mM bile salt concentration, respectively.

To better understand the impact of bile salt concentration and lipase activity on the rate constant (*k*) and extent (*C*_*f*_) of lipid digestion, correlation between these parameters was evaluated (Fig. 4).

Graphs A and B in Fig. 4 show that *C*_*f*_ is correlated with both bile salt concentration and lipase activity. The final extent increased because of both increasing bile salt concentrations and increasing lipase activities. However, lipase activity predominantly determines final lipid digestion as the larger increase of *C*_*f*_ indicates for identical bile salt concentrations in graph B. These results were further confirmed by lipase exhibiting the lowest *P*-value (0.0001) followed by bile (0.0008), and their interaction term which was defined as bilelip (0.0023) in the linear regression model of *C*_*f*_ (Table 3).

**Table 3:** Output of multiple linear regression with backwards elimination for final extent *C*_*f*_ with parameter estimates, standard error and their significance level (significance level at 0.10), *R*^2^ = 1.00.

Variable	Parameter estimate	Standard error	*F* value	*P* > *F*
Intercept	6.62	0.40	272.55	0.0005
Bile	1.09	0.08	188.88	0.0008
Lipase	0.92	0.04	639.04	0.0001
Bilelip	−3.23 × 10^−3^	3.31 × 10^−3^	95.13	0.0023
Lipase^2^	−5.65 × 10^−3^	0.33 × 10^−3^	284.74	0.0005
Bile^2^	−1.67 × 10^−2^	0.31 × 10^−2^	28.31	0.013

The superscript 2 means the square of the value of lipase or bile.

Graphs C and D in Fig. 4 show a more complex correlation between *k* and the varied digestive conditions. Both graphs show that both bile salt concentration and lipase activity influence the rate constant of hydrolysis rather than the isolated parameters. On the one hand, a declining trend of *k* was observed with increasing bile salt concentrations when lipase activity is 5 U/mL (Fig. 4C). On the other hand, a clear increasing trend was observed when lipase activity is 100 U/mL, while bile salt concentration did not seem to affect *k* at the intermediate lipase activity of 20 U/mL. Furthermore, low bile salt concentrations (2 mM) did not seem to affect the rate constant of digestion as much as the higher concentrations (10 and 20 mM) as trendlines are diverging more as the bile salt concentration increased (Fig. 4C). This was also illustrated by the significant differences among *k*-values presented in Table 2. Multiple linear regression revealed that solely the interaction term of bile salt concentration and lipase activity (bilelip) significantly influenced the rate constant *k* on a 10% significance level (Table 4).

**Table 4:** Output of multiple linear regression with backwards elimination for rate constant k with parameter estimates, standard error and their significance level (significance level at 0.10), *R*^2^ = 0.98.

Variable	Parameter estimate	Standard error	*F* value	*P* > *F*
Intercept	2.37 × 10^−2^	0.31 × 10^−2^	60.15	0.0002
Bile	−7.88 × 10^−4^	3.37 × 10^−4^	5.47	0.0579
Bilelip	6.03 × 10^−5^	0.41 × 10^−5^	215.88	<0.0001

## 4 Discussion

The present study aimed to evaluate lipid digestion kinetics under a range of bile salt concentrations and lipase activities since they represent the different life stages of broiler birds. Conditions in the gastric and small intestinal phase (e.g. changing pH) might affect emulsion microstructure and consequently lipid digestion kinetics (McClements, 2018). Therefore, microstructural evaluations of samples of TC 1 and 9, being the outer conditions of this study, were executed. Larger oil droplets observed in small intestinal digests of TC 9 can be contributed to depletion flocculation phenomena that occur as a result of higher bile salt concentrations. In general, bile salts are surface active components which lower the interfacial tension of oil droplets and thus increase emulsion stability in comparison to situations where no bile salts are present (Grundy and Wilde, 2021; Mun et al., 2007). Nevertheless, when the bile salt concentration exceeds a certain limit, typically higher than the critical micellar concentration, it might also negatively impact emulsion stability. In that case, coalescence occurs as a result of depletion flocculation (Jódar-Reyes et al., 2010). As exactly the same lysolecithin-based emulsifier was used for both TC 1 and 9, this phenomenon was completely attributed to the higher bile salt concentration for the case of TC 9 (Jódar-Reyes et al., 2010; Mun et al., 2007).

Furthermore, changing bile salt concentrations and lipase activities from lower to higher quantities in the context of lipid digestion evaluation in broilers at different ages significantly impacted the rate constant *k* and final extent *C*_*f*_ of lipid hydrolysis. Bile salts and lipase are essential compounds to efficiently digest lipids. Firstly, bile salts need to adsorb to the interface, displacing a fraction of emulsifiers originally present at the interface, which in turn facilitates lipase adsorption. Furthermore, bile salts can remove lipid digestion products, such as free fatty acids and monoacylglycerols, from the interface and aid to assemble them into mixed micelle structures. In context of the latter, bile salts are also facilitating lipase adsorption (Mun et al., 2007). Secondly, and obviously, lipase is needed and must be active to hydrolyse lipids. Reduced lipase activities, as in the case of young broilers and weaning piglets, have been proven to result in a lower extent of lipid hydrolysis (Gu and Li, 2003; Krogdahl, 1985). In this case, undigested lipids can remain at the end of the small intestine. This is unfavourable as research indicated that this results in enzymatic digestion disorders and eventually in overall malabsorption of nutrients (Knarreborg et al., 2002). Our research demonstrated the significantly lower extent of lipid digestion when simulating starter conditions in contrast to finisher conditions which is accordance with in vivo research. The coefficient of total tract apparent lipid digestibility of soybean oil almost doubles from starter to finisher birds (Tancharoenrat et al., 2013). Nevertheless, it has to be taken into consideration that the data of the current study only refers to lipid hydrolysis, while in vivo data is more complex also taking lipid micellarisation and absorption into account. In the current work, lipid digestion was incomplete in all TCs as high concentrations of oil were subjected to digestion while simultaneously applying lower bile salt concentrations and lipase activities.

Furthermore, the correlation analysis between *C*_*f*_ and the digestion conditions revealed that mainly lipase activity influenced the final extent. This is explained by the increasing enzyme-to-substrate ratio. This phenomenon is also observed in vivo in humans suffering from exocrine pancreatic insufficiency who typically secrete fewer lipase than healthy adults (Carrière et al., 2005; Keller, 2005). Moreover, our results also demonstrated the more complex interplay of bile salt concentration and lipase activity on the rate constant *k*. While bile salts promote digestion by adsorbing to the interface, thereby decreasing interfacial tension, displacing emulsifiers, and thus aiding lipase adsorption, an overdosage might inhibit lipid digestion. This overdosage is often described as the critical micellar concentration of bile salts. It is thought that when bile salts are present in relatively high concentrations compared to lipase activity levels, it completely covers the interface and so inhibits lipase adsorption to the interface (Borgström and Erlanson, 1973; Brownlee et al., 2010; Futami et al., 1982; Gargouri et al., 1983). Bile salt inhibition could not be overcome by insufficient lipase activity, in this case 5 U/mL. Nevertheless, when sufficient lipase is present, bile salts have a promoting effect on lipid digestion. Hence, to effectively digest lipids, sufficient amounts of both bile salts and lipase should be available in the GIT. This is further confirmed by the significantly higher *C*_*f*_- and *k*-values in case of TC 9. More importantly, our results strongly indicate the importance of adapting and distinguishing digestion conditions relevant for broilers in different life stages.

In this work, a total gastric digestion time of 2 h was considered. However, this duration is tending to relatively longer digestion times reported in vivo (Svihus, 2011; Svihus et al., 2002). Therefore, shortening of gastric digestion to 60 to 90 min might be more appropriate in future studies, especially when taking other macronutrients into account (e.g. protein and starch). Nevertheless, the current dataset remains unaffected by this as gastric lipase is lacking.

## 5 Conclusion

In vitro research into feed additives with the potential to promote lipid digestion in broiler chickens can be used to complement in vivo experiments. However, adapted and standardised protocols to simulate digestion in poultry are currently lacking*.* This paper specifically focussed on proposing appropriate in vitro lipid digestion conditions to mimic the GIT of broilers at 3 different life stages (i.e. starter, grower, and finisher). The final extent of lipid digestion (*C*_*f*_) mainly increased as an effect of increasing lipase activity, and to a lesser extent by increasing bile salt concentrations. Furthermore, sufficient amounts of bile salts and lipase activities resulted in the most efficient lipid digestions as reflected by reaction constant *k*. However, lipid digestion was inhibited by larger bile salt concentrations at critically low lipase activities. Thus, the present results clearly demonstrated significant effects of both bile salt concentration and lipase activity on lipid digestion kinetics. In other words, lipid digestion will be impacted by the life stage of broilers as this affects the secreted amounts of bile salt and lipase. Hence, it is strongly encouraged to use the suggested and appropriate digestive conditions to simulate the digestive tract of broilers and to employ different digestive conditions depending on the broilers’ life stage to be investigated.

## Author contributions

**Michels, D.**: Conceptualization, data curation, formal analysis, investigation, methodology, validation, visualization, writing - original draft; **Verkempinck, S.H.E.**: Conceptualization, methodology, supervision, writing - review & editing; **Panozzo, A.**: resources, writing - review & editing; **Vermeulen, K.**: resources, writing - review & editing; **Hendrickx, M.E.**: Data curation, writing - review & editing; **Thijs, L.**: Conceptualization, funding acquisition, project administration, resources, supervision, writing - review & editing; **Grauwet, T.**: Conceptualization, funding acquisition, project administration, supervision, writing - review & editing.

## Declaration of competing interest

We declare that we have no financial and personal relationships with other people or organizations that can inappropriately influence our work, and there is no professional or other personal interest of any nature or kind in any product, service and/or company that could be construed as influencing the content of this paper.
