# Supplementary material for: Importance of adapted digestion conditions to simulate in vitro lipid digestion of broilers in different life stages
Source: Anim Nutr. 2022 Oct 4;12:151–8. doi: 10.1016/j.aninu.2022.09.008 (PMC9842858; doi:10.1016/j.aninu.2022.09.008)
Supplement: Multimedia component 1 [file mmc1.pdf]

Supplementary data

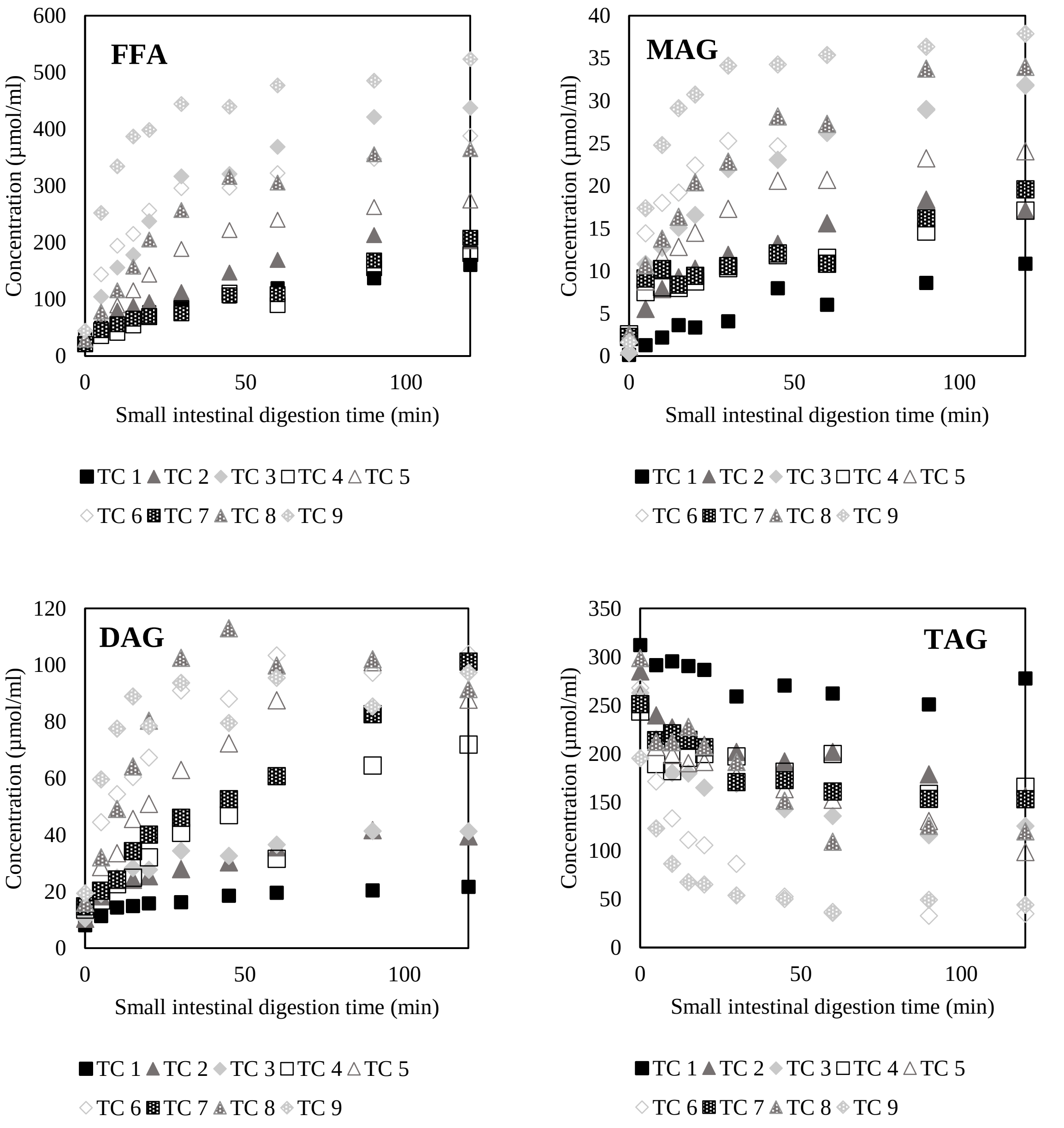

Fig. A. Concentration of lipid digestion products (FFA, MAG, DAG, and TAG) for all 9 test combinations (TC) (Table 1). Symbols represent experimental data: (■) 5 ,(▲) 20, and (◆) 100 U lipase activity per mL chyle. Fillings represent (full) 2, (empty) 10, and (speckled) 20 mM bile salt concentrations.
